# Arthralgia and myalgia associated with aromatase inhibitors: frequency and characterization in real-life patients

**DOI:** 10.3332/ecancer.2024.1697

**Published:** 2024-04-16

**Authors:** Natalia Camejo, Cecilia Castillo, Diego Santana, Lucia Argenzio, Dahiana Amarillo, Guadalupe Herrera, Maria Guerrina, Gabriel Krygier

**Affiliations:** 1 Oncology Service, Hospital de Clínicas Dr. Manuel Quintela, Montevideo 11600, Uruguay; 2 Oncology Service, Departmental Hospital of Soriano “Zoilo A. Chelle” – U.E 30. Soriano, 75000, Uruguay; 3 Department of Quantitative Methods, School of Medicine, Montevideo 11800, Uruguay

**Keywords:** breast neoplasm, aromatase inhibitor, adverse effects, arthralgia

## Abstract

**Introduction:**

Adjuvant treatment with aromatase inhibitors (AI) in oestrogen receptor-positive and/or progesterone receptor-positive breast cancer (BC) has been shown to increase overall survival. However, arthralgias and myalgias are common adverse effects in patients treated with AI.

**Objective:**

To evaluate the frequency and characteristics of arthralgias and myalgias in patients with early BC-treated adjuvantly with AI in the Mastology Unit of the Oncology Service of the Hospital de Clínicas and the Departmental Hospital of Soriano.

**Materials and methods:**

A prospective, cross-sectional and descriptive study was performed. A questionnaire was administered to patients to assess the presence and characteristics of arthralgias and myalgias associated with AI.

**Statistical analysis:**

‘Age’ was described with measures of central tendency and dispersion. Qualitative variables were presented in absolute and relative frequencies. Logistic models were used to evaluate the association between patient characteristics, tumour characteristics, treatment characteristics and the presence of pain. Results were presented by odds ratio and *p*-value, using R software (version 4.1.2) with a significance threshold of 5%.

**Results:**

83 patients were included, with a median age of 69 years. 75.9% presented arthralgias and/or myalgias related to treatment, with an average intensity of 5–7. 80.9% received non-steroidal anti-inflammatory drugs (NSAIDs), achieving satisfactory analgesia. The presence of arthralgias and myalgias was significantly associated with age and time since the last menstrual period (LMP), being more frequent in patients older than 50 years and those with more than 5 years since the LMP.

**Conclusion:**

Approximately 70% of the patients presented arthralgias or myalgias. These findings suggest a possible role of oestrogen withdrawal in its mechanism of development. Multidisciplinary and translational research is crucial to evaluate the ethology and therapeutic options for patients with AI-related arthralgia.

## Background

Cancer is a problem of significant importance in Uruguay’s epidemiologic panel. In fact, taking both sexes together, cancer is the second cause of death after cardiovascular diseases, accounting for almost a quarter (24.6%) of the total number of deaths registered in our country each year. Almost 16,179 new cases are registered annually and more than 7,995 Uruguayans die from this cause [1, 2].

As is observed worldwide, breast cancer (BC) in Uruguay is the most frequent cancer in women and also the main cause of death from cancer. Every year, about 1975 new cases are registered in our country and about 689 women die as a consequence of this disease [2]. According to our estimates, the lifetime risk of developing this cancer in Uruguay is 1 in 11 women [3].

It is widely known that adjuvant treatment with aromatase inhibitors (AI) for 5 years in oestrogen receptor and/or progesterone receptor-positive BC increases disease-free survival (DFS) and overall survival (OS) [4, 5].

AI are associated with joint and muscle symptoms, commonly referred to as AI-associated musculoskeletal syndrome. The prevalence of arthralgias ranges from 20% to 70% while the presence of myalgia ranges from 10% to 60% of patients treated with hormone therapy (HT). Musculoskeletal symptoms can deteriorate the quality of life of patients and lead to discontinuation of treatment [6, 7], a Dutch study showed that 74% of women treated with AI developed arthralgias and myalgias which significantly impacted their personal and family life, recreation and occupation [8]. In this context, one study reported a treatment discontinuation rate of 13% at 1 year and 21.2% at 3 years [6]. Recently a study reported that most Latina women with MC have medium or low levels of adherence to AI therapy [9].

The importance of the side effects associated with AIs intensified after these drugs were shown to improve both DFS and OS compared to tamoxifen in adjuvant BC treatment. This has led to AIs now being the treatment of choice in postmenopausal women with hormone receptor-positive early BC [10]. In addition, combining or continuing HT with AIs after 5 years of tamoxifen has also been found to prolong SVLE [11].

While arthralgias and myalgias are a known adverse effect of AI treatment little is known about the frequency of AI-related arthralgias and myalgias in patients with early MC in clinical practice, outside of clinical studies. Therefore, it is crucial to conduct research that deepens the understanding of the characteristics of AI-induced arthralgia in order to develop effective interventions.

The present study will investigate the frequency and characteristics of arthralgias and myalgias related to AI in patients with early MC attended at the Mastology Unit (UM) of the Hospital de Clínicas and the Departmental Hospital of Soriano and identify the demographic factors that could have an impact on the development of these adverse effects.

## Methods

### Primary objective

To evaluate the frequency and characteristics of arthralgias and myalgias in patients receiving adjuvant treatment with AI for early MS attended at the UM of the Oncology Service of the Hospital de Clínicas and the Departmental Hospital of Soriano in the period between 1 January 2015 and 31 December 2023; and to identify the demographic factors that could impact on the development of these adverse effects.

### Methods

This is a prospective, cross-sectional, descriptive study whose objective is to evaluate the frequency and characteristics of arthralgias and myalgias in patients with ER/RP-positive early BC attended at the UM of the Oncology Service of the Hospital de Clínicas and at the Departmental Hospital of Soriano in the period between 1 January 2015 and 31 December 2023, who have received adjuvant treatment with AI for at least 6 months.

Patients with rheumatoid arthritis, osteoarthritis, fibromyalgia, gout or any other form of arthritis were excluded.

A cross-sectional survey design was used to collect information from the participants at a single point in time. The questionnaire assessed the presence of joint or muscle pain, before and after initiation of treatment with Al.

The questionnaire included data on pain intensity as measured by the visual analog scale (VAS), its location and its interference with activities of daily living, sleep, enjoyment of life and mood. We also inquired about the analgesic treatment received and its efficacy.

Data regarding the date of diagnosis, date of last menstrual period (LMP), type of HT treatment received (anastrazole, letrozole or exemestane) and its duration were collected through a thorough study of clinical histories, maintaining the anonymity of the patients. An excel database was used to analyse the data, where each patient was assigned an identifying number.

After the consultation, the objectives of the study were explained to the patients who were candidates for inclusion in the study and they were invited to participate. All the patients signed an informed consent form, by which they agreed to participate in the study, and also authorised the use of the information arising from the study.

### Study variables

Variables related to the patient were included: age at diagnosis and current age; origin (Montevideo or the interior of the country, rural area); marital status; who she lives with, educational level and occupation.

### Tumour-related variables

Date of diagnosis, LMP, histologic type and grade; pathologic tumour size; axillary lymph node status; stage according to TNM classification; HER 2 status.

### Treatment-related variables

Type of breast and axillary surgery, type of systemic and radiant treatment, type of adjuvant HT (anastrazole vs. exemestane vs. letrozole) and duration.

For the analysis and reporting of these data, the anonymity of the patients was maintained.

AI-related arthralgias and/or myalgias were defined as any new or worsening joint and/or muscle pain after starting any AI. As mentioned above, joint and muscle pain was measured by VAS.

### Statistical analysis

The quantitative variable ‘age’ is described by its measures of central tendency and dispersion. The qualitative variables (origin, with whom she lives, educational level, marital status and stage) are presented by their absolute and relative percentage frequencies. To evaluate the association between the characteristics of the patient, the tumour, the type of treatment and the presence or absence of pain, logistic models were performed; the results are presented using the odds ratio with the *p*-value.

All analyses were performed in R software (version 4.1.2) considering a significance threshold of 5%.

### Ethical aspects

The proposed study was conducted in accordance with international ethical standards for biomedical research: ‘MERCOSUR Norms on Regulation of Clinical Studies’ and the ‘Declaration of Helsinski’, and with the research regulations approved by the National Ethics Commission in 2019.

Likewise, in accordance with the provisions of the Data Protection Law, Law 18.331, the data were handled anonymously and were not disclosed to third parties, this information being exclusively known by the patient and the treating medical team. Personal data were not used during the analysis of the data nor during the dissemination of the results of the present study.

## Results

Eighty-three patients were included, the median age at the time of the survey was 69 years (range 47–87). In relation to marital status, the majority (60.2% *n* = 50) were married or living with a partner. 30.1% (*n* = 25) lived alone, 42.2% with their children (*n* = 35) and 15.7% with their parents (*n* = 13). Regarding their origin, 13 people (15.7%) were residents of rural areas. The vast majority (96.4%) were treated with anastrazole. The remaining epidemiological and demographic characteristics of the patients and the treatment they received are shown in Table 1.

**Figure 1.**
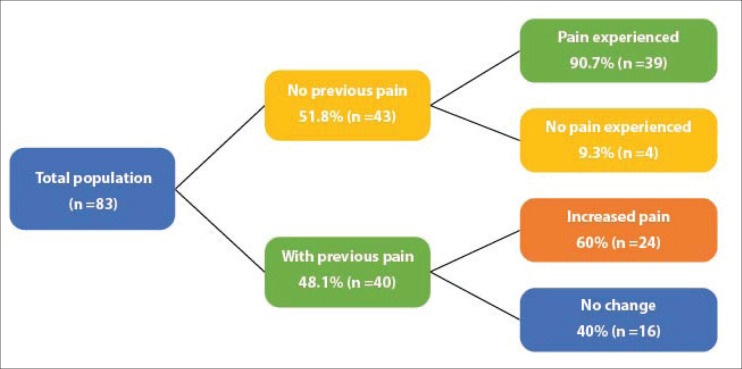
Frequency of arthralgias and myalgias.

Of the total patients, 75.9% (*n* = 63) had treatment-related arthralgias and/or myalgias (Figure 1). The averages for the intensity of arthralgias and myalgias for patients without previous pain were 2.5 and 3.5, respectively, while for patients with previous pain the average pain for the intensity of arthralgias and myalgias was 3 and 1.5, respectively.

### Joint pain

45.8% of the patients included (*n* = 38) presented joint pain after the start of treatment with HT, the most frequent topographies being: 27.3% fingers and knees, 21.2% fingers and ankles and 15.2% fingers. Of these, 63.9% (*n* = 21) suffered an increase in joint pain. The rest of the data are shown in Table 2.

Joint pain was statistically significantly associated with age, occupation and time elapsed since the LMP, being more frequent among patients >50 years old, retired and who had their LMP more than 5 years ago. Table 3.

### Muscle pain

42.2% of the patients included (*n* = 35) presented muscle pain after HT treatment with the most frequent topographies being: 38.5% upper and lower limbs, 29.2% spine and 26.2% lower limbs. Eighty percent of these (*n* = 24) suffered an increase in muscle pain. The rest of the data are shown in Table 4.

Muscle pain was statistically significantly associated with age, occupation and time elapsed since the LMP, being more frequent among patients >70 years old, retired and who had their LMP more than 5 years ago. Table 5.

It was not possible to relate arthralgias and myalgias to the type of AI received since the vast majority of patients received anastrazole, Table 1.

Significant findings regarding topography and pain intensity. Myalgias occurred most frequently in the upper and lower limbs (35.5%) and spine (35.5%). As for arthralgias, the most commonly affected areas were the hands/knees (27.9%) and spine (18.6%) (Table 6).

In addition, a considerable proportion of patients experienced moderate to severe pain in both myalgias and arthralgias. Specifically, 69.8% of patients with arthralgias and myalgias reported a maximum pain intensity between 5 and 7 on a scale of 1 to 10 (with 0 being no pain at all and 10 being the worst pain imaginable) and 27.9% rated their pain as very severe, with intensities between 8 and 10 (Table 7).

Regarding analgesic treatment: 80.9% (*n* = 51) received non-steroidal anti-inflammatory drugs (NSAIDs) and 19.1% (*n* = 12) with weak opioids, achieving satisfactory analgesia (average 8, with 0 not relieving and 10 completely relieving).

Regarding the impact of joint and muscle pain on daily activities and mood, we observed that arthralgias tended to interfere more markedly with various activities and emotional aspects than myalgias. Although the overall impact of both types of pain is moderate, arthralgias had a greater impact on general activity, mood, walking, and work, both inside and outside the home, with scores ranging from about 2.3 to 2.5 on a scale of 0 to 10. In comparison, myalgias showed slightly less impact, with average scores below 1.3 in these same areas. Sleep and enjoyment of life were also affected, although to a lesser degree, by both types of pain (Table 8).

## Discussion

In our investigation, we conducted a comprehensive assessment of AI-related arthralgias and myalgias as perceived by the patients, as well as their impact on daily activities and mood. Of the patients, 75.9% experienced AI-related arthralgias or myalgias (47% experienced them after starting treatment and 29% had an increase in the intensity of arthralgias or myalgias already present at the time of starting treatment). Of these patients, 67.4% reported a moderate average pain intensity. These figures are in line with international studies, where the prevalence of arthralgia varies between 30% and 70% and that of myalgias between 20% and 50% [12, 13]; as well as that evidenced outside clinical trials, in real-life studies [14].

It is important to consider that in our study we excluded patients with pre-existing conditions such as rheumatoid arthritis, osteoarthritis, fibromyalgia, gout or any other form of arthritis, which could lead to an underestimation of the impact of AI treatment on joints and muscles. In addition, since our study was based on self-reports, there is a possibility of classification bias due to patient perception. Despite these limitations, our findings provide valuable information on the experience of BC patients on AI treatment and underscore the need for effective and personalised pain management strategies.

Controlling for clinical and demographic factors, we observed that older patients, retired patients and those with a longer interval since their LMP experienced arthralgias and myalgias more frequently. This pattern could be partially explained by the relationship between time since menopause and residual oestrogen levels. Women who have recently entered menopause may have higher oestrogen levels, so the initiation of AI treatment, which drastically reduces these levels, could intensify arthralgia symptoms. Recent studies suggest that this abrupt decrease in oestrogen caused by AIs may be a key factor in the development of arthralgia [15].

Given the diversity in the pathophysiological patterns and mechanisms of AI-induced arthralgias, a detailed analysis of their manifestations is essential to better understand how they affect patients’ functionality and quality of life. The intensity of these arthralgias can vary significantly, and their impact on patients’ daily lives depends largely on how severe the pain is. For example, high intensity joint pain in key joints such as the hands or knees and spine as evidenced in our study can considerably limit patients’ autonomy and mobility. An average of 67.4% of the included patients experienced moderate pain. This finding highlights the importance of considering not only the presence of arthralgias, but also their intensity, when developing specific therapeutic strategies. An approach that takes into account both the location and severity of pain is essential to improve pain management and, therefore, the quality of life of these patients.

In this context, it is critical for health care professionals to be aware of these symptoms from the onset of AI treatment. Early identification of arthralgia may allow for more timely interventions, such as medication adjustments, pain management therapies or physical rehabilitation strategies, which could prevent an exacerbation of symptoms and improve patients’ quality of life.

Considering that myalgias secondary to AI treatment may present variations in their location and underlying pathophysiological mechanisms, it is crucial to examine the specific manifestations of myalgias in different muscle areas. Understanding the topography of these myalgias is essential to assess their impact on patients’ function and quality of life, as well as to guide pain management and pain relief strategies. In addition, it is important to note that the intensity of muscle pain varies significantly, differentially affecting patients. For example, muscle pain in areas of high mobility such as the upper and lower limbs may have a more limiting effect on daily activity compared to other areas. In our study, we analysed in detail the regions most affected by myalgias in patients treated with AI, finding that the most common locations included the upper and lower limbs (35.5%) and the spine (35.5%) (Table 6). A considerable proportion of patients experienced moderate to severe pain (Table 7), emphasising the importance of considering both the location and severity of pain when developing treatment plans. This detailed information is crucial for the development of targeted and effective interventions to improve patients’ well-being during and after AI treatment.

In relation to daily activities and mood, our findings indicate that, although arthralgias and myalgias generally have a moderate to low impact, arthralgias seem to more significantly affect the daily life of patients treated with AI. This aspect is particularly relevant, as it highlights the need for comprehensive pain management that encompasses both joint and muscle pain. An effective approach to pain management would not only improve the quality of life of these patients but could also positively influence their adherence and response to BC treatment.

Finally, these results underscore the importance of clear and ongoing communication between physicians and patients about the potential side effects of AI treatment. By educating patients about the signs and symptoms of arthralgias and myalgias and establishing a pain management plan from the onset of treatment, treatment adherence and, ultimately, long-term outcomes can be significantly improved. This is particularly crucial as premature discontinuation of AI treatment due to unmanaged side effects can negatively affect BC prognosis.

Our study provides a comprehensive view on the frequency and characteristics of arthralgias and myalgias associated with AI treatment in patients with BC. We found that a significant proportion of patients, specifically 75.9%, experienced these side effects, with a moderate impact on their quality of life and daily activities. The prevalence and intensity of joint and muscle pain highlight the need for personalised and effective pain assessment and management in these patients.

The impact of arthralgias and myalgias on daily life and mood, although generally moderate, emphasises the importance of clinical care that considers both the severity of pain and its location. This is particularly relevant for those patients who have recently entered menopause, who may experience more intense symptoms due to hormonal changes associated with treatment.

## Conclusions

In conclusion, our findings underscore the importance of personalised pain management strategies as well as ongoing and effective physician-patient communication to improve adherence to AI treatment and ultimately to improve health and quality of life outcomes for patients with BC. This study also highlights the need for future research exploring innovative therapeutic strategies to address these challenges in the management of BC.

## Conflicts of interest

The authors deny conflicts of interest.

## Funding

The study was not financed.

## Author contributions

**Concepción:** Natalia Camejo, Cecilia Castillo, Diego Santana, Lucía Argenzio, Dahiana Amarillo and Gabriel Krygier.

**Design:** Natalia Camejo, Cecilia Castillo, Diego Santana, Lucía Argenzio and Dahiana Amarillo.

**Execution:** Natalia Camejo, Cecilia Castillo, Diego Santana, Lucía Argenzio and Dahiana Amarillo.

**Analysis:** Natalia Camejo, Cecilia Castillo, Guadalupe Herrera **Interpretation of results:** Natalia Camejo, Cecilia Castillo, Dahiana Amarillo, Guadalupe Herrera and María Guerrina.

**Editorial staff:** Natalia Camejo, Cecilia Castillo, Dahiana Amarillo, Guadalupe Herrera and María Guerrina.

**Critical review:** Gabriel **Krygier**

## Figures and Tables

**Table 1.** Epidemiological and demographic characteristics of the patients included in the study and the treatment received (*n* = 83).

Variables	(*n*)	%
Age category, years		
≤50	9	10.8
51–70	57	68.7
>70	17	20.5
Marital status		
Married or living with a partner	50	60.2
Divorced	14	16.9
Single	3	3.6
Widow	16	19.3
Educational level		
Completed elementary school	10	12
Incomplete high school	18	21.7
High school completed	49	59
Tertiary	6	7.2
Occupation		
Housewife	10	12.0
Retired or pensioner	30	36.1
Employee	43	51.9
Time since LMP		
≤5 years	17	20.4
5–10 years	32	38.6
>10 years	34	41.0
Stadium		
EI	21	25.3
IBD	46	55.4
EIII	16	19.3
Received treatment with QT		
No	32	38.6
Yes	51	61.4
Received treatment with RT		
No	15	18.1
Yes	68	81.9
Received trastuzumab treatment		
No	66	79.5
Yes	17	20.5
Type of HT		
IA	80	96.4
Letrozole	3	3,6
HT time		
≤3 years	30	36.1
4–5 years	15	18.1
>5 years	38	45.8

**Table 2.** Presence and topography of arthralgias (*n* = 83).

Variable		(*n*)	%
**Presence of arthralgias prior to initiation of treatment (*n* = 83)**			
	No	50	60.2
	Yes	33	39.8
Topography of arthralgias			
	Column	5	6.1
	Fingers of the hands	4	15.2
	Fingers and back	9	12.1
	Fingers and knees	7	27.3
	Fingers and ankles	1	21.2
	Knees	5	3.0
Presence of arthralgias after initiation of treatment (*n* = 50).			
	No	12	24.0
	Yes	38	76.0
Topography of arthralgias	Hip	2	5.3
	Column	6	15.8
	Fingers of the hands	9	23.7
	Fingers and back	10	26.3
	Fingers and knees	10	26.3
	Ankles	1	2.6

**Table 3.** Characteristics according to the presence of treatment-related arthralgias.

	Arthralgia *n* (%)		
	No	Yes	*p*
Age at diagnosis <0.001			
≤50	7 (58.3)	2 (3.4)	
51–70	5 (41.7)	40 (67.8)	
>70	0 (0.0)	17 (28.8)	
Marital status 0.27			
Married/partnered	7 (58.3)	37 (62.7)	
Divorced	4 (33.3)	7 (11.9)	
Single	0 (0.0)	2 (3.4)	
Widow	1 (8.3)	13 (22.0)	
Occupancy <0.001			
Housewife	4 (33.3)	5 (8.5)	
Employee/independent	8 (66.7)	20 (33.9)	
Retired	0 (0.0)	34 (57.6)	
Educational level 0.46			
Tertiary	0 (0.0)	1 (1.7)	
UTU	1 (8.3)	3 (5.1)	
High school completed	5 (41.7)	35 (59.3)	
Incomplete high school	5 (41.7)	12 (20.3)	
Completed elementary school	1 (8.3)	8 (13.6)	
Time since LMP <0.001			
≤5 years	10 (83.3)	7 (11.9)	
5–10 years	2 (16.7)	26 (44.1)	
>10 years	0 (0.0)	26 (44.1)	
Stage 0.43			
I	3 (25.0)	16 (27.1)	
II	9 (75.0)	34 (57.6)	
III	0 (0.0)	9 (15.3)	
Received treatment with QT 0.05			
No	2 (16.7)	28 (47.5)	
Yes	10 (83.3)	21 (52.5)	
Type of breast surgery 1			
CC	8 (66.7)	36 (61.0)	
Mastectomy	4 (33.3)	23 (39.0)	
Type of axillary surgery 0.72			
BGC	2 (16.7)	16 (27.6)	
VAG	10 (83.3)	42 (72.4)	
Received treatment with RT 0.21			
No	4 (33.3)	9 (15.3)	
Yes	8 (66.7)	50 (84.7)	
Received treatment with trastuzumab 1			
No	10 (83.3)	51 (86.4)	
Yes	2 (16.7)	8 (13.6)	
Type of HT 1			
IA	12 (100)	56 (94.9)	
Letrozole	0 (0.0)	3 (5.1)	
Time of HT 1			
≤3 years	4 (33.3)	22 (37.3)	
4–5 years	2 (16.7)	10 (16.9)	
>5 years	6 (50.0)	27 (45.8)	

**Table 4.** Presence and topography of myalgias (*n* = 83).

Variable		(*n*)	%
**Presence of myalgias prior to treatment initiation (*n* = 83)**			
	No	53	63.9
	Yes	30	36.1
Topography of myalgias			
	Column	5	16.7
	Lower limbs	12	40.0
	Upper limbs	2	6.7
	MMSS and MMII	11	36.7
			
			
Presence of myalgias after initiation of treatment (*n* = 50).			
	No	18	34.0
	Yes	35	66.0
Topography of myalgias			
	Column	19	29.2
	Upper limbs	4	6.2
	Lower limbs	17	26.2
	MMSS and MMII	25	38.5

**Table 5.** Characteristics according to the presence of treatment-related myalgias.

	Myalgias *n* (%)		
	No	Yes	*p*
Age at diagnosis 0.007			
≤50	1 (6.7)	8 (15.4)	
51–70	14 (93.3)	27 (51.9)	
>70	0 (0.0)	17 (32.7)	
Marital status 0.84			
Married/partnered	10 (66.7)	29 (55.8)	
Divorced	3 (20.0)	8 (15.4)	
Single	0 (0.0)	2 (3.8)	
Widow	2 (13.3)	13 (25.0)	
Occupancy <0.001			
Housewife	2 (13.3)	6 (11.5)	
Employee/independent	12 (80.0)	14 (26.9)	
Retired	1 (6.7)	32 (61.5)	
Educational level 0.08			
Tertiary	1 (6.7)	0 (0.0)	
UTU	2 (13.3)	2 (3.8)	
High school completed	8 (53.3)	28 (53.8)	
Incomplete high school	4 (26.7)	13 (25.0)	
Completed elementary school	0 (0.0)	9 (17.3)	
Time since LMP <0.001			
≤5 years	8 (53.3)	9 (17.3)	
5–10 years	7 (46.7)	17 (32.7)	
>10 years	0 (0.0)	26 (50.0)	
Stage 0.09			
I	8 (53.3)	12 (23.1)	
II	6 (40.0)	32 (61.5)	
III	1 (6.7)	8 (15.4)	
Received treatment with QT 0.87			
No	7 (46.7)	23 (44.2)	
Yes	8 (53.3)	29 (55.8)	
Type of breast surgery 0.05			
CC	12 (80.0)	27 (51.9)	
Mastectomy	3 (20.0)	25 (48.1)	
Type of axillary surgery 0.74			
BGC	3 (20.0)	14 (27.5)	
VAG	12 (80.0)	37 (72.5)	
Received treatment with RT 0.27			
No	1 (6.7)	12 (23.1)	
Yes	14 (93.3)	40 (76.9)	
Received treatment with trastuzumab 1			
No	12 (80.0)	42 (80.8)	
Yes	3 (20.0)	10 (19.2)	
Type of HT 1			
IA	15 (100.0)	50 (96.2)	
Letrozole	0 (0.0)	2 (3.8)	
HT time 0.35			
≤3 years	4 (26.7)	18 (34.6)	
4–5 years	1 (6.7)	10 (19.2)	
>5 years	10 (66.7)	24 (46.2)	

**Table 6.** Topography of treatment-related arthralgias and myalgias.

Variables	*N*	%
Topography of arthralgias (*n* = 43)		
Fingers of the hand	4	9.3
Column	8	18.6
Knee	3	7
Hip	3	7
Fingers and spine	10	2.3
Fingers and knee	12	27.9
Fingers and ankle	1	2.3
Ankles	1	2.3
Topography of myalgias (*n* = 43)		
Lower limbs	14	35.5
Upper limbs	1	2.3
Lower and upper limbs	14	35.5
Column	14	35.5

**Table 7.** Intensity of arthralgias and myalgias.

Variables	*N*	%
Arthralgias		

Maximum intensity 1–4	1	2.3
Maximum intensity 5–7	30	69.8
Maximum intensity 8–10	12	27.9

Average intensity 1–4	14	32.5
Average intensity 5–7	29	67.4
Average intensity 8–10	0	0

Lower intensity 1–4	3	6.9
Lower intensity 5–7	4	9.3
Lower intensity 8–10	0	

Myalgias		

Maximum intensity 1–4	1	2.3
Maximum intensity 5–7	30	69.8
Maximum intensity 8–10	12	27.9

Average intensity 1–4	14	32.6
Average intensity 5–7	29	67.4
Average intensity 8–10	0	0

Lower intensity 1–4	39	90.7
Lower intensity 5–7	4	9.3
Lower intensity 8–10	0	0

**Table 8.** Interference of joint and muscle pain on activities and mood (0 does not interfere and 10 interferes completely).

Type of activity	Arthralgias	Myalgias
General activity	2.3	1.3
State of mind	2.5	1.2
Ability to walk	2.4	1.2
Normal work (inside and outside the home)	2.3	1.1
Dream	2.3	1.1
Enjoy life	2.2	1.1
